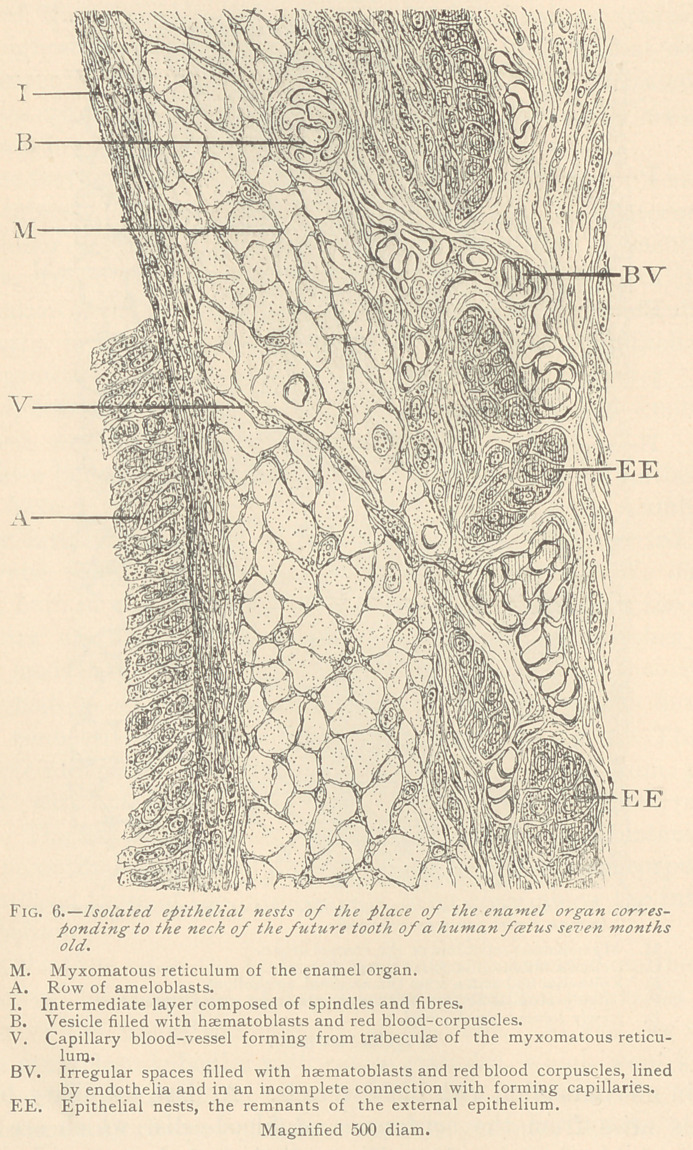# Contributions to the History of Development of the Teeth

**Published:** 1887-05

**Authors:** Carl Heitzmann, C. F. W. Bödecker


### Independent Practitioner.

VOL. VIII. MAY, 1887. NO. 5.

### Original Communications.

NOTE.—No paper published or to be published in another journal will be accepted for this department. All papers must be in the hands of the Editor before the first day of the month preceding that in which they are expected to appear. Extra copies will be furnished to each contributor of an accepted original article, and reprints, in pamphlet form, may be had at the cost of the paper, press-work and binding, if ordered when the manuscript is forwarded. The Editor and Publishers are not responsible for the opinions expressed by contributors. The journal is issued promptly, on the first day of each month.

### CONTRIBUTIONS TO THE HISTORY OF DEVELOPMENT OF THE TEETH.

BY CARL HEITZMANN, M. D., AND C. F. W. BÖDECKER, D. D. S., M. D. S.

### I. HISTORY OF THE DEVELOPMENT OF THE ENAMEL.

The fact has long since been established that the first step toward the formation of teeth in the human subject is the appearance of a furrow behind the lips, corresponding to the cartilaginous jaws, about the sixth week of intra-uterine life. At this time a trace of cancellous bone is usually formed around the cartilage. The epithelium covering the furrow is stratified. In vertical sections through the upper and lower jaws of an embryo of this age, we observe a depression surrounded on either side by an elevation, which is bounded toward the lips by a shallow ridge, and toward the tongue by a varying number of alternating depressions and shallow elevations—the floor of the oral cavity. The embryonal tissue corresponding to the furrow is characterized by an accumulation of medullary corpuscles, as well as by a cord-like formation, the corpuscles being in form mostly spindle-shaped.

At the end of the second month of intra-uterine life, the furrow is deepened, and lined with stratified epithelium, the deepest layer consisting of columnar epithelia which are plainly marked. The labial wall of the furrow may exhibit secondary elevations indicative of papillæ. From the bottom of the furrow, or one of its walls, a prolongation of epithelia takes place, which originally is composed of columnar epithelia, but later appears in the shape of a solid epithelial point or cord, whose borders are composed of columnar, and whose interior is filled with cuboidal epithelia. All these changes take place between the third and fourth month of intra-uterine life, and can be observed best in frontal sections of the jaws.

No accurate time can be fixed for these formations, since frontal sections of a three-month embryo exhibit different stages of epithelial prolongations, as well as epithelial cords which have reached a certain depth, and are broadened at their ends like a cup or bell jar (Tomes) surrounding a knob-like accumulation of medullary corpuscles, which is the first trace of the dentine papilla.

The epithelial peg or cord is either stretched out, or it runs a more or less wavy course. Not infrequently knobs or buds grow out from it with a peculiar concentric arrangement of the epithelia. The epithelial cord, in some instances, may be short, almost immediately broadening after its origin from the surface, or it may be of varying, nay considerable length, taking an oblique, or devious course to such an extent that the cord runs parallel with the cup-shaped enlargement, and is inserted into the latter at an acute angle. It also occurs that the main epithelial cord runs almost parallel with the outer surface, giving off a branch interiorly for the formation of the temporary tooth, while the cord itself extends further, terminating bluntly at its distal end. Close below the furrow, from the main epithelial cord a lateral cord sprouts out, likewise more or less knobbed, which, as is generally admitted, represents the first trace of a permanent tooth.

If we examine one of these cup-shaped enlargements, we observe a distinctly marked border composed of columnar epithelia, whereas the interior of the cup is filled with medullary corpuscles, which, in the centre, present the so-called stellate reticulum. At the end of the fourth and the beginning of the fifth month, we invariably find some epithelial cords, which, at their interior ends, are broadened, and contain a distinctly marked stellate reticulum. (Fig. 1.)

The first question to be entered into is: whence comes the myxomatous tissue known as the stellate reticulum in the interior of the cup? According to our present knowledge of the tissues of the mammalian organism, we are entitled to call the stellate reticulum a myxomatous tissue, which is a variety of connective tissue. This tissue occurs most extensively in the embryonal organism, and remains throughout life in the fully developed body only in a limited number of organs, such as the pulp of a tooth, the lymph ganglia, and the so-called adenoid tissue, which properly ought to be called lymph tissue, and is distributed throughout the mucous membranes, especially during the early periods of life. Unless we assume that the enamel organ is a tissue entirely different from all others entering into the structure of the body, we must call it myxomatous connective tissue.

Those who strictly adhere to the teachings of Thiersch and Waldeyer, will be loath to admit that epithelium can ever change into connective tissue. Previous researches, however, have led us to the conviction that such a transformation is by no means impossible. The thyroid gland, for instance, is originally composed of alveoli lined by epithelia. Shortly after birth the epithelium, however, is replaced by a medullary or lymph tissue. The whole central nervous system (the brain and spinal cord) originates from the embryonal epiblast, which is strictly epithelial in nature. Nevertheless, nobody will maintain that the central nervous system, so richly supplied with blood-vessels, is an epithelial structure, except in the lining of the ventricles of the brain, and the central canal of the spinal cord. There may be advocates of the exclusive nature of epithelial tissue, who might think of an immigration of medullary corpuscles between the epithelia of the enamel organ for the benefit of the formation of the stellate reticulum, but there is not the least indication of such a process in any of our specimens. On the contrary, we can prove a gradual transformation of the epithelia into myxomatous tissue.

In the third month of intra-uterine life, we observe, inside of the epithelial cup of the enamel organ, a zone entirely occupied by medullary corpuscles, and even in the fourth and fifth month such a gradual transition is distinctly traceable. (Fig 2.)

Those who still adhere to the cell doctrine will never be able to understand how medullary tissue arises from epithelia. According to our views, however, there exist no individual cells, but layers of protoplasm, in which the living matter is distributed in a reticular arrangement. Every particle of the living matter is able to grow from the size of a minute granule to that of a solid lump, in which a differentiation afterwards takes place into a peripheral protoplasm containing comparatively little living matter, and a central body, termed nucleus, with a larger amount of living matter.

The inner epithelia, at the period mentioned above, exhibit augmented nuclei and small glistening granules near the fold, corresponding in position to the neck of the future tooth. The more we turn to the centre of the cup, the more will we be struck by the presence of glistening homogeneous lumps in the epithelia, until we have reached the centre of the cup, where we observe that epithelium has been transformed into a number of such lumps in a regular arrangement, which reminds us of their origin from previous epithelia. The original epithelia gradually become enlarged, and at last are split up into a number of medullary corpuscles. As a rule, this process of transformation is most marked in the original epithelia at the portion directed toward the stellate reticulum, whereas, in that portion nearest the papilla, the epithelial character may still be preserved. (Fig. 3.)

The medullary corpuscles first assume a spindle shape, constituting the intermediate layer (*stratum intermedium*). The innermost spindles are in connection with a comparatively coarse network, representing the first trace of the stellate reticulum. The trabeculæ of this reticulum are composed of solid or vacuoled spindles, enclosing spaces which appear to be filled with a distinctly reticulated protoplasm, holding central nuclei. The latter exhibit a varying number of coarser granules, the so-called nucleoli.

Not infrequently the intermediate layer is missing, which affords the best opportunity for observing the gradual transition of the homogeneous globules arising from the epithelia into nucleated protoplasmic bodies, and at last into the myxomatous reticulum.

Changes similar to those described take place in the central portions of the external epithelia, and, as it seems, even preceding those of the inner. Thus the original columnar bodies of the outer epithelium are reduced to a row of cuboidal epithelia, as seen in Fig. 2, E. E. The medullary corpuscles are slightly enlarged, their nuclei, at first plainly visible, are likewise split up into a delicate reticulum, and both become infiltrated with a myxomatous basis-substance. The peripheral portions of the original medullary corpuscles, on the contrary, are solidified into nucleated formations of living matter, representing the stellate reticulum proper. The meshes of the myxomatous tissue in the stellate reticulum are originally small, and correspond in size to the medullary corpuscles, from which they arose. The corpuscles of the stellate reticulum are mostly solid. Later, several medullary corpuscles are required for the formation of a large field of basis-substance. The original stellate reticulum, in this view, must fall back to an embryonal or medullary tissue before changing into a more perfect myxomatous tissue, such as we observe from the end of the fifth month of fœtal life up to the full development of the enamel. (Fig. 4.)

FIG. 4.—*Stellate reticulum, or myxomatous tissue, of the enamel organ of a human fœtus of five months of intra-uterine life.*

Magnified 1200 diam.

Toward the end of the fourth, and the beginning of the fifth month, the stellate reticulum is composed of nucleated protoplasmic bodies, with a varying number of branching and inter-connecting offshoots. With low powers of the microscope, the basis-substance in the meshes, enclosed by the corpuscles and their offshoots, appears to be homogeneous and structureless. The highest powers, however, reveal in this basis-substance the presence of a delicate reticular structure, even without the addition of any reagent. This structure has arisen by a direct transformation of the original medullary corpuscles into basis-substance. In the highest development of the stellate reticulum, such as seen in the seventh and eighth month of fœtal life, the nucleated corpuscles are more slender, and the reticulum is composed mainly of delicate branching and inter-connecting fibres.

The further changes of the external epithelium are of considerable interest. While about the fourth month of intra-uterine life the inner portions of the external epithelium are, as mentioned above, transformed into medullary tissue and participate in the formation of the myxomatous enamel organ, a single row of cuboidal epithelia is left. From the remains of this external epithelium, a new growth takes place of a markedly centrifugal character. By a multiplication of the epithelial elements, solid buds and knobs are formed, well known to previous observers. (Fig. 5.)

These buds are at first in continuity with the external epithelium, and have a distinct layer of columnar and a varying number of inner layers of cuboidal epithelia. They are characterized by a brownish color, common to all epithelial formations. We observe that, both in the central portions of these buds and along the original row of the external epithelium, a transformation takes place into medullary corpuscles, the same as we observe toward the myxomatous enamel organ. This medullary tissue develops into connective tissue of a decidedly fibrous character. Thus we observe numerous interruptions in the external epithelium, partly filled with medullary and partly with fibrous connective tissue. The latter is in direct connection with the myxomatous reticulum, or this connection is established by spindle-shaped corpuscles, resembling those of the intermediate layer close above the internal epithelium.

At the time when the buds sprout from the external epithelium, an active new formation of blood-vessels and blood corpuscles takes place in the immediate vicinity of the buds. At first, we notice large protoplasmic bodies with coarse granules, which were known to Theodore Schwann, in 1839, by the name of blood-cells. With the increase of the size of these bodies the granules likewise become coarser and assume the properties of the so-called hæmatoblasts. These grow up to the size of red blood corpuscles, and we not infrequently encounter in the bays between the buds, groups of hæmatoblasts, or fully developed blood corpuscles, apparently isolated

FIG. 5.—*Budding of the external epithelium of the enamel organ of a human fœtus seven months old.*

M. Myxomatous reticulum of the enamel organ.

C. Delicate fibrous connective tissue.

E. Epithelial bud arisen from the external epithelium.

P. Large protoplasmic body filled with glistening coarse granules.

B. Newly formed blood-vessel.

Magnified 500 diam.

and in no connection with blood-vessels. At last, capillary blood-vessels arise from the conference of blood-cells, which are filled with red blood corpuscles. The splitting of the external epithelium into isolated buds and nests of an epithelial character, is especially marked near the neck of the future tooth. (Fig. 6.) At this place the amount of myxomatous enamel organ in a sevenmonth fœtus, is usually small, since a great quantity of it has already been transformed into enamel tissue. But even here, a few small and isolated epithelial nests are seen, surrounded by a large number of capillary blood-vessels, filled with blood corpuscles. It is obvious that all these blood-vessels are newly formed, and indeed we can trace the formation of blood-vessels in this situation, step by step. Even the myxomatous trabeculæ of the enamel organ will participate in the formation of capillary blood-vessels. We have seen closed spaces, or vesicles, sprung from the basis-substance of the myxomatous tissue, filled with hæmatoblasts and red blood corpuscles, partly in connection with already formed or forming capillaries. No doubt the living matter enclosed in the basis-substance has grown into hæmatoblasts. This process is indicated by the appearance of either coarsely granular or compact glistening nuclei in the meshes of myxomatous reticulum.

Wherever we observe epithelial nests, they are invariably enlarged toward the enamel organ by spindles and fibres of an intermediate layer. Their scarcity and diminutiveness at the place corresponding to the neck of the tooth, indicates that they are completely transformed into medullary tissue. Considering the fact that at the end of the intra-uterine development the enamel organ is nearly exhausted, and the enamel which is formed up to that time is comparatively thin, there is good reason for the assumption that the medullary tissue sprung from the previous external epithelium is the source for the completion of the enamel, such as we observe upon temporary teeth when they emerge from their socket.

(TO BE CONTINUED.)

## Figures and Tables

**Fig. 1. f1:**
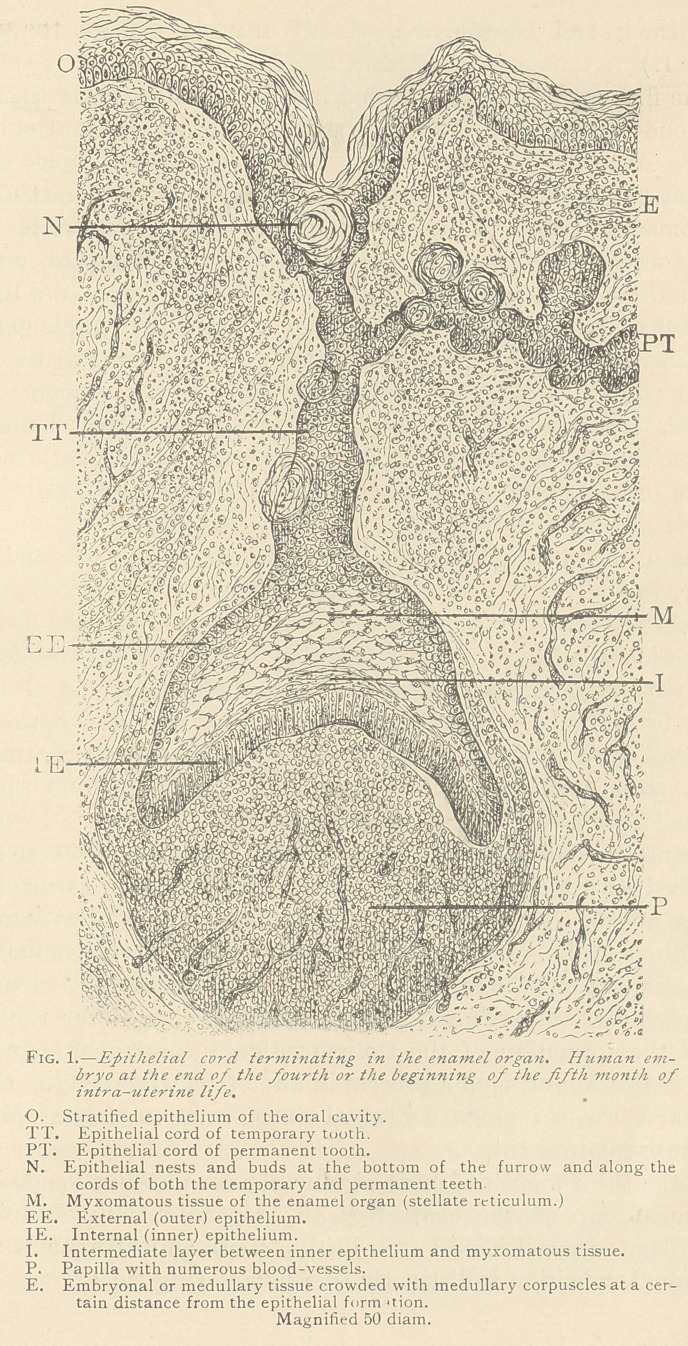


**Fig. 2. f2:**
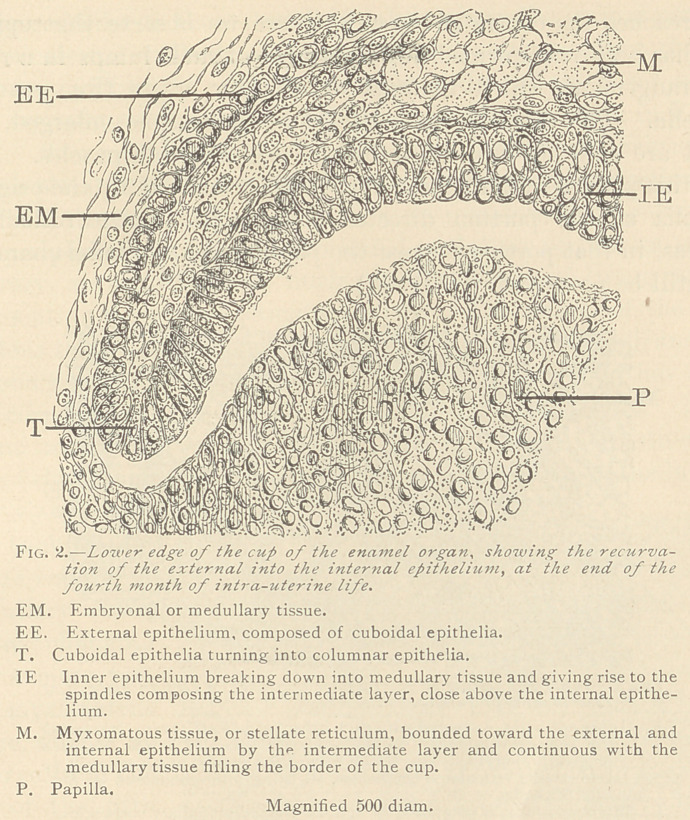


**Fig. 3. f3:**
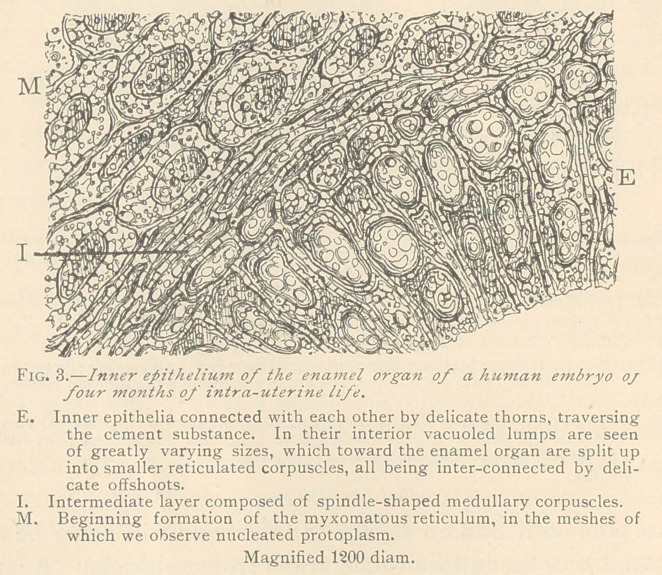


**Fig. 4. f4:**
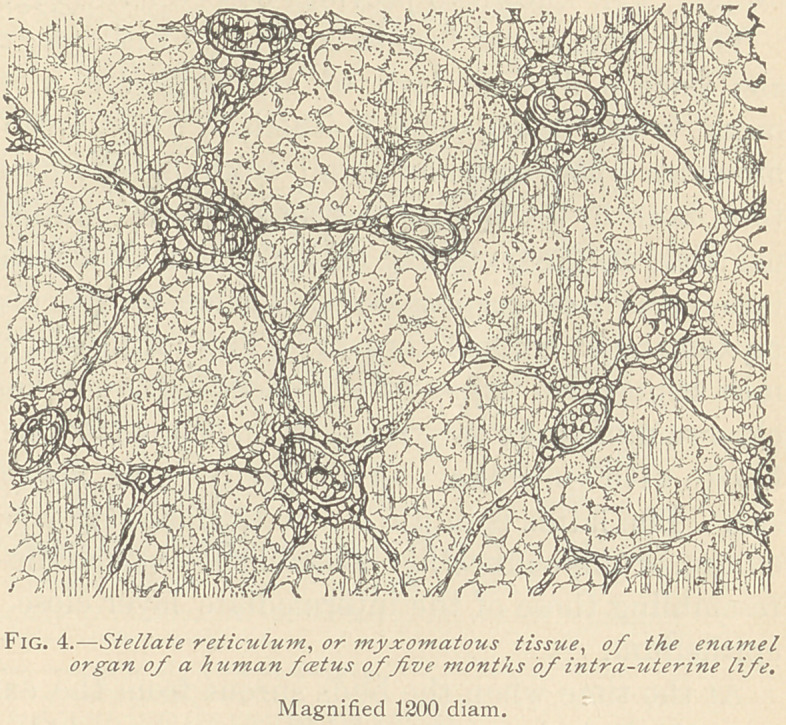


**Fig. 5. f5:**
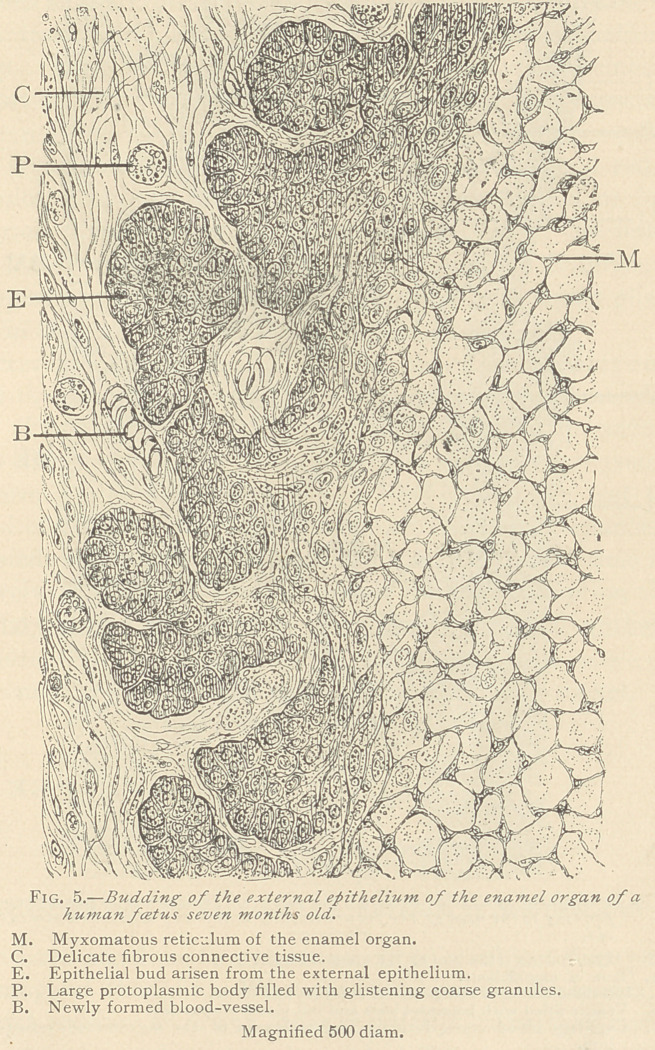


**Fig. 6. f6:**